# Dermatophyte-Selective Imidazole-Thiosemicarbazides: Potent In Vitro Activity Against *Trichophyton* and *Microsporum* with No Anti-*Candida* Effect

**DOI:** 10.3390/ijms26199437

**Published:** 2025-09-26

**Authors:** Agata Paneth, Katarzyna Dzitko, Adrian Bekier, Nazar Trotsko, Katarzyna Suśniak, Anita Ciesielska, Piotr Paneth

**Affiliations:** 1 Chair and Department of Organic Chemistry, Medical University of Lublin, 20-093 Lublin, Poland; 2 Department of Molecular Microbiology, Faculty of Biology and Environmental Protection, University of Lodz, 90-237 Lodz, Polandadrian.bekier@umed.lodz.pl (A.B.);; 3 Department of Pharmaceutical Microbiology, Medical University of Lublin, 20-093 Lublin, Poland; 4 Institute of Applied Radiation Chemistry, Faculty of Chemistry, Lodz University of Technology, 90-924 Lodz, Poland

**Keywords:** imidazole, thiosemicarbazide, dermatophytes, antifungal activity, docking study

## Abstract

Dermatophytes are highly infectious pathogenic fungi that colonize keratinized tissues like skin, hair, and nails, causing superficial infections such as tinea capitis, onychomycosis, tinea corporis, and tinea pedis in humans and animals. In immunocompromised patients, they may invade deeper tissues and organs, leading to severe or life-threatening conditions if untreated or inadequately managed. While most infections respond to topical antifungals, some require complex treatment and show resistance to standard therapies. Therefore, novel antifungal agents are needed. We investigated the antidermatophytic activity of imidazole-thiosemicarbazides against *Microsporum canis*, *Trichophyton* spp., and *Chrysosporium* spp. using the broth microdilution method, comparing results to ketoconazole and amphotericin B through minimal inhibitory concentration (MIC), half-maximal inhibitory concentration (IC_50_), and selectivity index (SI). Iodine- and bromine-substituted compounds showed the strongest activity, with MICs of 15.15 (IC_50_ < 1 μM; SI > 213) and 73.46 μg/mL (IC_50_ < 1 μM; SI > 846) against *T. tonsurans*, and 3.87 (IC_50_ = 7.21 μM; SI > 29.6) and 7.38 μg/mL (IC_50_ = 11.06 μM; SI = 76.6) against *M. canis*. In silico analysis revealed interactions with α-keratin and lanosterol-14-α demethylase (the azole target enzyme), suggesting enhanced drug retention and action. These findings support these compounds as promising leads for further antifungal development.

## 1. Introduction

Dermatophytes are among the most common pathogenic fungi and a major cause of superficial fungal infections worldwide, affecting large populations globally [1,2,3,4,5,6]. According to the Global Burden of Disease (GBD) studies, skin and subcutaneous diseases collectively rank among the leading causes of non-fatal disease burden worldwide (4th overall in 2010 and 2013), underscoring the population-level impact of superficial mycoses, including dermatophytosis [7,8]. In line with this burden, WHO estimates based on the IHME’s GBD 2024 report indicate approximately 650 million fungal skin infections worldwide, of which about half are dermatophytosis (tinea/ringworm) [4]. Other GBD analyses—depending on the metric used and the reference year—report higher totals (~1.6–1.7 billion), but also indicate ringworm as the largest component of the burden of fungal skin infections [7,8]. As keratinophilic organisms, dermatophytes infect the keratinized host tissues including the skin, hair, and nails, leading to inflammatory responses and clinical entities collectively termed dermatophytosis, commonly referred to as tinea (ringworm) [6,9]. These filamentous fungi can also colonize human hosts without causing disease [1,2]. According to national ambulatory care surveys (NAMCS/NHAMCS), dermatophyte infections accounted for 4,981,444 outpatient visits in the United States between 2005 and 2014. The associated direct medical costs were estimated at approximately USD 821 million (2017 USD) [10]—equivalent to ≈USD 845 million in 2019 dollars, as cited in a later review [3]. More broadly, an economic analysis conducted by CDC authors estimated that fungal diseases overall were associated with direct medical costs of roughly USD 7.5 billion in 2019 [11].

**Figure 1:**
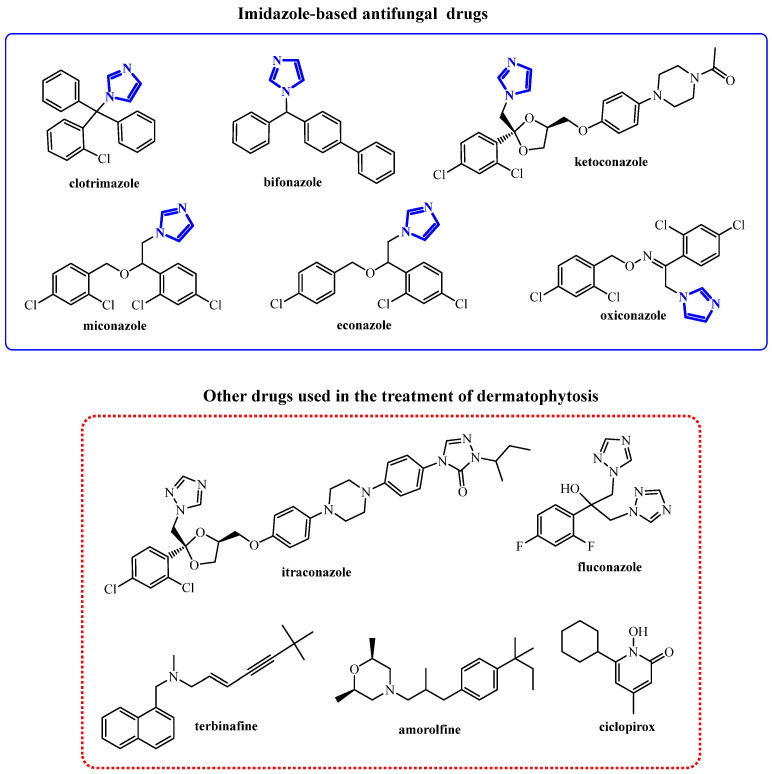
Antifungal drugs used in the treatment of dermatophytosis.

From a clinical perspective, dermatophyte infections are classified according to the anatomical site affected. Onychomycosis (tinea unguium; fungal nail infection) is typically the most difficult form to cure, followed by tinea capitis. Tinea pedis (commonly known as “athlete’s foot”), in turn, is the most common with a prevalence of ~3% in the general population [12], but is substantially higher in specific high-risk groups (e.g., patients with diabetes, athletes, soldiers) [13], whereas tinea corporis (fungal infection of the body) and tinea cruris (superficial fungal infection of the groin and buttocks region) are reported less frequently. Although most dermatophyte infections are not life-threatening and respond well to standard topical therapy, some require prolonged and complex regimens, including combined topical and systemic treatment [14,15]. Topical treatments typically include imidazole-based drugs (e.g., clotrimazole, ketoconazole, miconazole) or allylamines (e.g., terbinafine, naftifine); in addition to ciclopirox or amorolfine which are used especially for nail diseases [14,16,17]. In systemic therapy, in turn, oral antifungal drugs like fluconazole, itraconazole, and terbinafine are widely used [18] (Figure 1).

In addition to costs associated with treatment, many reports suggest that drug resistance, including overexpression of efflux pumps, drug detoxification, mutation in drug targets, and modification of cellular metabolism to overcome drug effects, is an emerging problem, particularly terbinafine resistance linked to mutations in the squalene epoxidase gene, which worsens treatment outcomes [3,19,20]. Initially observed in India, this resistance has spread globally, including Europe, Iran, Japan, China, and the United States. Beyond terbinafine, a first-line treatment for dermatophytosis, the most common dermatophyte species, such as *Trichophyton rubrum* and *Trichophyton mentagrophytes*, have also developed considerable resistance to widely used azole antifungals like fluconazole, voriconazole, and itraconazole. The main driver of azole resistance is the enhanced efflux of the drug from fungal cells, although decreased drug uptake and structural alterations at the target site also contribute [3]. Additionally, many systemic antifungal agents have notable adverse-effect profiles, including hepatotoxicity, gastrointestinal symptoms, allergic reactions, hematologic effects, and potential drug–drug interactions [21,22,23]. While topical treatments such as amorolfine 5% and ciclopirox 8% generally cause only mild side effects—mainly localized skin irritation and hypersensitivity—they tend to be less effective when used alone, especially in conditions like onychomycosis [16,17,24]. Therefore, there is a clear need to develop new therapeutic strategies combining high efficacy with reduced toxicity and specifically targeting the emerging antifungal resistance in dermatophytes [6,15].

Research strategies aiming for novel antifungals are being pursued in three primary directions. The first one focuses on the resource-intensive development of new classes of compounds that would exhibit the desired high antifungal activity, often targeting novel molecular targets or multi-target effects, making these compounds more effective against resistant strains. The second research strategy involves fine-tuning existing antifungal compounds to improve their effectiveness, spectrum of activity, or reduce toxicity. The last approach refers to drug repurposing or re-evaluation of known bioactive compounds for novel therapeutic applications.

**Figure 2:**
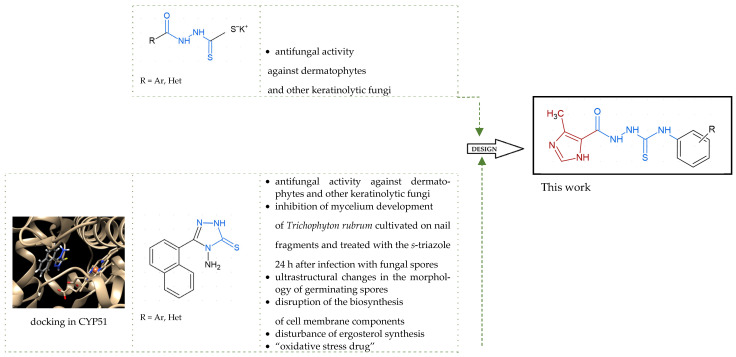
Overview of the results of previous studies [25] and design of novel antidermatophytic hybrid compounds.

Our previous investigation with potassium salts of *N*-acylhydrazinecarbodithioates and their cyclic *s*-triazole analogues culminated in the discovery of their potential as lead structures for novel antidermatophyte and other keratinolytic fungi agents development (Figure 2) [25]. The compounds were effective against anthropophilic dermatophyte *Trichophyton rubrum* which is responsible for the majority of dermatophyte-associated infections globally [2,9]. Most of them were also effective against zoophilic dermatophyte *M. canis* that frequently infects both humans and animals, making it one of the most encountered dermatophytes in clinical practice [26,27]. Additionally, using a representative *s*-triazole we examined in vitro effects of such chemical structures on the morphology of *Trichophyton rubrum*. The microscopic observations revealed inhibition of mycelium development of *Trichophyton rubrum* cultivated on nail fragments and treated with the *s*-triazole 24 h post-inoculation with fungal spores, as well as ultrastructural changes in the morphology of germinating spores. Finally, the RNA-seq analysis revealed that this compound, like undecanoic acid and acriflavine, disrupts the biosynthesis of cell membrane components and can lead to decreased ergosterol levels. Furthermore, due to its ability to upregulate the expression of some genes in *T. rubrum* whose products are related to the oxidative stress response, this compound, like undecanoic acid, amphotericin B, and itraconazole, has been defined as an “oxidative stress drug.”

With the hypothesis that the introduction of the imidazole moiety into the thiosemicarbazide scaffold as privileged substructure could be exploited for system optimization, series of known anti-*Toxoplasma gondii* compounds with an imidazole-thiosemicarbazide scaffold (IMI-TSCs) [28,29] were resynthesized and validated as a pharmacophore for antifungal activity against dermatophytes (Figure 2). The initial screening indicated that some of them are promising candidates. In silico studies confirmed that these compounds have the potential to interact with both α-keratin and lanosterol-14-α demethylase (CYP51), which is consistent with the initial design.

## 2. Results and Discussion

### 2.1. Rationale

As mentioned in the Introduction, although dermatophytes are not usually life-threatening and respond well to currently available topical antifungal agents, some dermatophyte infections can cause severe or unusual infections, especially in patients with immune suppression [30,31,32,33,34]. In addition, some dermatophyte infections require complex treatment regimens, and there is an increasing trend towards chronic infections, characterized by persistent or recurrent episodes lasting six months or longer despite treatment [35,36,37]. Despite the development of dermatophytosis treatment science and technology, it is still treated with commercially available topical or oral azole-based antifungals with many side effects [38]. Furthermore, resistance in dermatophytes and other superficial fungal pathogens, primarily of *Candida* species, increased alarmingly [39]. Approximately 20% to 25% of the world’s population is affected by superficial mycoses, primarily caused by dermatophytes, and the prevalence is likely to change with changing migration patterns, increased tourism, and changes in socioeconomic conditions [40]. Therefore, novel antifungal compounds for the treatment of superficial fungal infections are a key area of research.

Among various heterocyclic substituents, the imidazole ring is a versatile building block in medicinal chemistry, found in various commercially available pharmaceuticals, insecticides, herbicides, and other bioactive compounds [41]. Anticancer, anti-inflammatory, antirheumatic, antihistaminic, antiallergic, antiasthmatic, antioxidant, antihypertensive, antiobesity, antidiabetic, antiulcer, antidepressant, anticonvulsant, analgesic, anthelmintic, antiprotozoal, antimalarial, antiamoebic, antitubercular, antiaging, anticoagulant, antiviral, antibacterial, and antifungal properties may all be produced chemically by altering the imidazole scaffold [42]. Antifungal activity is one of these applications and one of the main areas of research studies for imidazole compounds.

Previously, several imidazoles with the pharmacophore thiosemicarbazide scaffold were created by our group and tested in vitro for their anti-*Toxoplasma gondii* efficacy. Several compounds with selective pressure and more potent inhibitory effect than sulfadiazine, trimethoprim, and a combination dose of these drugs on actively replicating forms of the parasite, i.e., tachyzoites, were identified [28,29]. Further in vivo studies on a mouse model confirmed their effectiveness against both acute toxoplasmosis caused by highly virulent *Toxoplasma gondii* type I strain as well as the latent bradyzoite form (i.e., cyst-forming type II *Toxoplasma gondii* strain), which contributes to chronic disease. Moreover, these compounds have been effective against *Toxoplasma gondii* infection in the brain due to their blood–brain barrier penetrability. Finally, in vivo studies confirmed their good pharmacokinetics and safety profile [43].

Inspired by promising results for IMI-TSCs as prospective chemotherapeutics, the current framework was designed to estimate their potential utility as antifungal agents. For this purpose, the hybrid molecules with optimal solubility under antimicrobial assay **1**–**10** (Table 1) were resynthesized according to a one-step synthetic procedure described in detail elsewhere [28,29], and then their antifungal efficacy against dermatophytes and non-dermatophyte species such as *Candida* spp. was tested in a preliminary in vitro antimicrobial screening.

### 2.2. In Vitro Antifungal Activity

Target IMI-TSCs **1**–**10** were tested for minimum inhibitory concentration (MIC) values in vitro against the reference dermatophyte strains, including *Trichophyton tonsurans*, *Trichophyton rubrum*, *Trichophyton mentagrophytes* (formerly *Trichophyton interdigitale*), *Microsporum canis*, as well as keratinophilic fungi *Aphanoascus keratinophilus* (formerly *Chrysosporium keratinophilum*), *Chrysosporium queenslandicum*, *Arthroderma pannicola* (formerly *Chrysosporium pannicola*), and *Chrysosporium tropicum*. *Trichophyton* species are pathogenic and major causative agents of dermatophytosis. Among them, *Trichophyton rubrum* is the most common cause of dermatophytosis worldwide and is known for infecting the skin, nails, and hair. It is an anthropophilic fungus that typically causes chronic infections such as tinea pedis (athlete’s foot) and onychomycosis (nail infection). *Trichophyton tonsurans* is also an anthropophilic fungus that causes tinea capitis (scalp infection), especially common in certain populations, and is characterized by endothrix hair invasion. In contrast, *Trichophyton mentagrophytes* is a zoophilic fungus capable of causing inflammatory reactions and kerion formation on the scalp. While often considered contaminants when isolated, *Chrysosporium* spp. can also be pathogenic. These opportunistic pathogens primarily cause superficial infections such as skin infections and onychomycosis but can also lead to rare, sometimes severe systemic infections in patients with compromised immune systems, such as bone marrow transplant recipients or individuals with chronic granulomatous disease. These systemic infections have a high mortality rate. Zoophilic dermatophyte *M. canis*, in turn, frequently infects both humans and animals, making it one of the most encountered dermatophytes in clinical practice.

For these in vitro studies, ketoconazole was used as the reference drug, and amphotericin B, a broad-spectrum polyene, was included as a mechanistically distinct control. Mechanistically, ketoconazole inhibits lanosterol 14α-demethylase (CYP51), thereby blocking ergosterol biosynthesis and exerting primarily fungistatic effects, whereas amphotericin B binds ergosterol and forms membrane pores, leading to increased permeability, ion leakage, and rapid fungicidal activity. From a clinical point of view, ketoconazole is a well-established antifungal agent for treating *Trichophyton* infections, with oral therapy reserved for severe or resistant cases and topical therapy useful for localized skin infections. Amphotericin B also has antifungal activity against dermatophytes, including *Trichophyton* species, although it is not a first-line treatment. Its use is typically reserved for recalcitrant, resistant, or invasive *Trichophyton* infections, such as Majocchi’s granuloma caused by multidrug-resistant *T. rubrum*, rather than common superficial dermatophytosis. Although reported clinical cases indicate that *M. canis* isolates show full resistance to amphotericin B, ketoconazole’s antifungal activity is well documented for treating *M. canis* infections. Treatment susceptibility data for infections caused by *Chrysosporium* are limited but suggest some responsiveness to antifungal drugs, including amphotericin B and ketoconazole.

The MIC values of **1**–**10** were tested with reference to the broth microdilution assay, according to the European Committee on Antimicrobial Susceptibility Testing (EUCAST; E. Def 9. 3. 2) guidelines [44] with adaptations for dermatophytes as previously described [45,46]. The MICs were defined as the lowest concentration of the compound to totally inhibit (minimal inhibitory concentration 100%, MIC_100_) the visual growth of fungal cells compared to that of a drug-free control at 34 °C for 72 h incubation. A summary of MICs is presented in Table 1. The inhibitory concentration for 50% inhibition of fungal growth (IC_50_) and the selectivity index (SI) calculated by CC_30_/IC_50_ are presented in Table 2.

The biological activity data show that the antidermatophytic activity of the IMI-TSCs is fungal species-selective rather than broad-spectrum, except for the analogue with the aliphatic 2-chloroethyl group **10** that was found to be ineffective against all fungi studied. These results indicate that the aryl substituents of **1**–**9** enhance antifungal activity against dermatophytes through specific molecular interactions, such as, for example, π-π interactions with molecular targets that disrupt essential fungal processes, whereas the alkyl substituent of **10** lacks such interactions, resulting in no activity. Generally, for **1**–**9**, no direct correlation between the substitution pattern or electronic effect of substituents at the phenyl ring related to antidermatophytic activity was found, thereby indicating that the subtle electronic and/or steric variations caused by substituents, directly influencing the compounds’ physicochemical properties, interaction mechanisms with fungal targets, and species-specific susceptibilities, determine antifungal activity. As shown in Table 1, compounds with *meta* and *para* electron-donating methyl (**2**, **3**) and electron-withdrawing nitro (**5**, **6**) substitution exhibited inhibitory activity against *Trichophyton tonsurans* comparable to or exceeded that of amphotericin B. Furthermore, compound **3** demonstrated antifungal activity against *Trichophyton tonsurans* superior to ketoconazole. Regarding the *Microsporum canis* strain, the *meta*-bromine **7** and *meta*-iodine **8** analogues showed antifungal activity comparable to or better than ketoconazole.

Generally, the most sensitive strain to the tested compounds was *T. tonsurans* with MIC ranges of <0.29 to 15.15 μg/mL, except for *o*-nitro **4** (MIC = 98.81 μg/mL) and *m*-bromine **7** (MIC = 73.46 μg/mL) analogues. The activity of *o*-methyl **1** (MIC = 2.93 μg/mL), *m*-methyl **2** (MIC = 1.43 μg/mL), *p*-methyl **3** (MIC < 0.29 μg/mL), *m*-nitro **5** (MIC = 1.59 μg/mL), and *p*-nitro **6** (MIC = 3.65 μg/mL) analogues against *T. tonsurans*, one of the primary agents responsible for tinea capitis infections around the world [2,47], was superior to that of other compounds, indicating that electronic effect of the substituent is not favoured. In contrast, the antifungal activity of **4**–**9** with an electron-deficient benzene ring was more potent against *Ch. queenslandicum* and *Ch. pannicola* strains than that of the methyl compounds **1**–**3**, highlighting the importance of electron-withdrawing substitution. *T. rubrum*, *Ch. keratinophilum*, and *M. canis* strains were moderately sensitive or even resistant to tested IMI-TSCs; however, for the latter one, MIC values of 3.87 and 7.38 μg/mL were obtained for the best-acting analogues *m*-iodine **8** and *m*-bromine **7**, respectively. Surprisingly, the compounds inhibited the growth of *M. canis*, although they showed weak activity against *Trichophyton* species. With respect to the specificity of IMI-TSCs toward pathogens and mammalian cells, in all cases, the obtained IC_50_ values against sensitive dermatophyte strains were lower than the cytotoxic effect (SI > 1), indicating the selectivity in antifungal activity (Table 2).

To probe the role of the imidazole moiety at the N1-position of 4-arylthiosemicarbazide scaffold on antidermatophytic activity, structural analogues of representative model compounds **3**, **5**, **6**, **8** with a more lipophilic cyclopentane ring were then obtained. As summarized in Table 1, **11**–**14** showed weak activity across the dermatophytes tested, with the notable exception of *Chrysosporium tropicum*, which remained highly susceptible (MICs: 1.61–6.08 μg/mL). This loss of potency upon imidazole replacement supports the imidazole as a privileged substructure in this chemotype for antidermatophytic activity.

Subsequently, IMI-TSCs with antidermatophytic activity **1**–**9** were evaluated for their antifungal activity against reference strains of *Candida* spp., including *Candida albicans*, *Nakaseomyces glabratus* (*formerly Candida glabrata*), *Candidozyma auris* (formerly *Candida auris*), *Pichia kudriavzevii* (formerly *Candida krusei*), and *Candida parapsilosis*. *Candida* spp. are opportunistic yeasts that constitute part of the normal human microbiota. Under certain conditions, such as immunosuppression, dysbiosis, or disruption of epithelial barriers, these commensals can transition into a pathogenic state, leading to clinically significant infections. *Candida albicans* is the predominant etiological agent in cutaneous and mucosal candidiasis; however, other species, including *Candida glabrata* and *Candida auris*, are increasingly recognized as clinically relevant pathogens. Although the majority of *Candida* infections are confined to the skin and mucous membranes, the presence of predisposing factors can facilitate hematogenous dissemination, leading to candidemia and subsequent multi-organ involvement. Multidrug-resistant species, most notably *Candida auris*, represent an escalating global health threat. Preventive interventions, particularly in vulnerable patient populations, remain essential to mitigating the incidence and severity of Candida infections. As summarized in Table 3, although the compounds have a bactericidal effect against *Candida* spp. (MBC/MIC ≤ 4), the MIC values indicated their mild or moderate bioactivity.

### 2.3. Molecular Docking

Azole antifungal agents act by inhibiting the fungal enzyme lanosterol 14-alpha-demethylase, which is crucial for converting lanosterol to ergosterol in fungal cell membranes. As ergosterol is a vital component of the fungal cell membrane, its disruption leads to increased permeability and ultimately, fungal cell death. This mechanism of action makes azoles effective against various fungal infections. Preliminary results obtained by our group suggest that the molecular mechanism of antidermatophyte action of the *s*-triazole (Figure 2) is similar to that of azole antifungal agents; this compound interferes with the biosynthesis of cell membrane components and may lead to a decrease in ergosterol levels and, consequently, to the inhibition the growth of *T. rubrum* on nail samples during drug exposure [25]. Furthermore, luliconazole, an imidazole-based antifungal agent, is reported to have a broad spectrum of activity against pathogenic fungi, particularly dermatophytes of the *Trichophyton* genus [53]. Although its exact mechanism of action is currently unknown, it is thought that it binds well to nail α-keratin and, like other azole antifungal agents, inhibits ergosterol synthesis [54].

Based on these data, in silico studies were performed to explore the potential for combined keratinophilic effect along with the interactions at the azole antifungal drug-target site.

**Figure 3:**
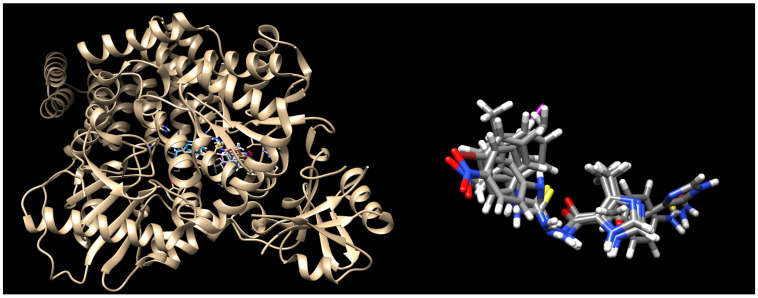
Binding mode of the IMI-TSCs **1**–**9** into α-keratin (PDB ID 4XIF). Nitrogen atoms are blue, oxygen atoms are red, sulphur atoms are yellow, and iodo atoms are magenta.

To assess the binding of the IMI-TSCs **1**–**9** to the α-keratin, following the docking studies presented by Hassan and co-workers [53], the crystal structure deposited in the Protein Data Bank under PDB code 4XIF [55] was selected. The best docking scores are listed in Table 4. The best binding poses are presented in Figure 3.

Docking results reveal that all the ligands bound tightly to α-keratin and all of them overlap well with the binding mode of the co-crystallized ligand. The N3 nitrogen atom of the imidazole head group of **1**–**9** interacts through H-bonds with Thr921 in a bidentate fashion, while its NH group serves as hydrogen donor for the oxygen atom of the amide group of Asn838. The binding mode of **1**–**9** is further stabilized by H-bond contacts between the carbonyl oxygen atom and Gly331, between three NH donor groups of the thiosemicarbazide skeleton and Asn838, Thr922, Phe837, as well as by several hydrophobic interactions with surrounding residues. *Some of them*, e.g., Gln839, Phe868, His921, Thr921, Thr922, are identical to those reported for the native ligand as well as to those predicted for luliconazole [54].

**Figure 4:**
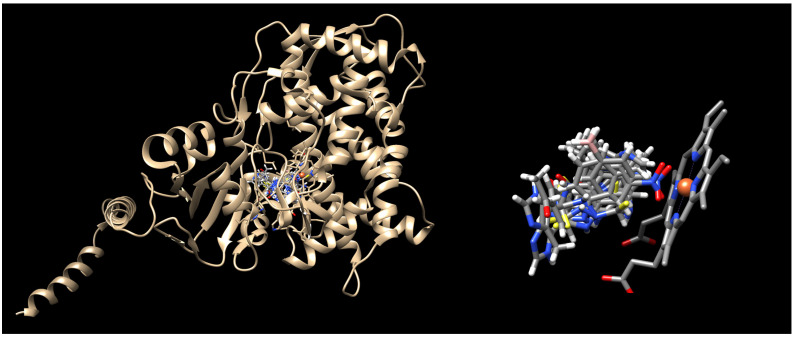
Binding mode of the IMI-TSCs **1**–**9** into lanosterol-14-α demethylase (PDB ID 5HS1). Nitrogen atoms are blue, oxygen atoms are red, sulphur atoms are yellow, nitrogen groups are pink, and the iron ion is orange.

To elucidate the binding mode of IMI-TSCs **1**–**9** at the lanosterol 14-α-demethylase active site, the crystal structure under PDB code 5HS1 [56] was selected. As presented in Figure 4, the ligands bind to the active site in a similar fashion to the co-crystalised voriconazole, yielding docking scores more favourable compared to the native ligand (Table 4). The imidazole ring of the ligands projects towards the phenolic hydroxyl group of Tyr140 or Tyr126 close enough to serve as hydrogen donors for H-bond interactions. The water-mediated polar interactions between the NH groups of the thiosemicarbazide core and the water molecule 726 further stabilise the binding of the ligands with the entry channel. Further docking analysis gives similar hydrophobic interaction networks with the catalytic domain of lanosterol-14-α demethylase enzyme (e.g., Phe134, Leu380, Leu383), as reported for the co-crystalised voriconazole.

Thus, based on the docking simulations, a combined keratinophilic effect along with molecular interactions with lanosterol-14-α demethylase are expected for IMI-TSCs, making them suitable as lead structures for further studies.

## 3. Materials and Methods

### 3.1. Chemistry

The IMI-TSCs **1**–**10** and the cyclopentane-thiosemicarbazides **11**–**14** were resynthesized according to a one-step synthetic procedure described in detail in Refs. [28,29,57], respectively. Briefly, ethanolic solution of the corresponding carboxylic acid hydrazide (0.001 mol) and an equimolar amount of the corresponding isothiocyanate was heated under reflux for 10–30 min. After cooling, the formed precipitate was collected by filtration, washed with diethyl ether, dried, and crystallized from ethanol. All commercial reactants and solvents were purchased from either Alfa Aesar (Kandel, Germany) or Tokyo Chemical Industry Co. (Tokyo, Japan) with the highest purity and used without further purification. Melting points (Gallenkamp MPD 350.BM 3.5 apparatus; Sanyo, Japan) and NMR spectra (Bruker Avance 300 MHz instrument with DMSO-*d*_6_ as solvent and TMS as an internal standard) for the compounds **1**–**14** were found in accordance with literature data [28,29,57].

### 3.2. Fungal Strains Used in This Study

The reference dermatophyte strains, including Trichophyton tonsurans CBS 118.65, Trichophyton rubrum CBS 120358, Trichophyton interdigitale CBS 124408, Microsporum canis CBS 11348, Chrysosporium keratinophilum CBS 104.62, Chrysosporium queenslandicum CBS 280.77, Chrysosporium pannicola CBS 116.63, and Chrysosporium tropicum CBS 171.62 from Westerdijk Fungal Biodiversity Institute (formerly CBS-KNAW Collections), the Netherlands, were used in this study. Strains were cultivated on Sabouraud agar (BTL, Bielany Wrocławskie, Poland) slants for 14 days at 28 °C. Colonies were covered with 5 mL of sterile water supplemented with 0.1% Tween-20 (P2287, Sigma, Oakville, ON, USA). The microconidia were carefully rubbed with a sterile wooden stick, filtered using Falcon 40 µm Cell Strainer (Corning, New York, NY, USA), and then transferred and germinated into 20 mL of YG medium (BTL) containing 0.5% yeast extract (Y1625, Sigma) as well as 2% glucose (G7021, Sigma) and cultivated for 3 h at 34 °C with agitation. Next, according to the European Committee on Antimicrobial Susceptibility Testing (EUCAST) (E. Def 9. 3. 2) [44] guidelines, the cell density of inocula was adjusted using a validated auto-calibrating turbidimeter, assuring a 0.5 McFarland standard [58,59]. Then the suspension was diluted 1:10 in sterile distilled water to yield 1–5 × 10^5^ CFU/mL.

### 3.3. Antifungal Activity Assays

Antifungal susceptibility tests were performed using the broth microdilution assay according to the European Committee on Antimicrobial Susceptibility Testing (EUCAST) (E. Def 9. 3. 2) [44] guidelines, with adaptations for dermatophytes [45,46]. Briefly, stock solutions of the tested compounds were two-fold diluted with RPMI-1640 (with 2% glucose, L-glutamine, without sodium bicarbonate, buffered at pH 7.2, R7388, Sigma) from 1000 to 7.8 µM (final volume 100 µL) in flat bottomed, clear 96-wells plate. The final DMSO (D4540, Sigma) concentration did not exceed 1% and had no influence on the growth of microorganisms. Then, a volume of 100 µL of standardized germinated conidia suspension (1–5 × 10^5^ CFU/mL) was added to each well. Microtiter plates were incubated at 34 °C in a moist, dark chamber for 72 h. Endpoints were defined using white-light microscopy as the lowest concentration of the compound resulting in total inhibition of growth compared to the growth in the control wells containing only quality control and no tested agents. All evaluations were performed in triplicate.

### 3.4. Resazurin Microtiter Assay

The resazurin microtiter assay plate method was used as previously described [60]. Briefly, clear 96-wells plate were prepared as described above (Section 3.3 Antifungal Activity Assay) and were covered, sealed with parafilm, and incubated at 34 °C in a moist, dark chamber for 7 days. Following, 30 µL of 0.02% resazurin (R7017, Sigma) solution was added to each well, and the plates were further incubated for 24 h. Changes in the sample color from blue to pink indicated the fungal growth. Plates were read with covered lids, and both 570 nm and 600 nm from the bottom using the multi-mode microplate reader SpectraMax^®^ i3 (Syngen, Tainan, Taiwan). The results were transformed to the percentage of viability compared to untreated cells, using the equation:

$$fungal\;growth\;\left[\%\right]=\frac{\left(A_{600/0}\times A_{570/S}\right)-\left(A_{570/0}\times A_{600/S}\right)}{\left(A_{600/0}\times A_{570/100}\right)-\left(A_{570/0}\times A_{600/100}\right)}\times 100\%$$

where *A* is the measured absorbance at a given wavelength, 0 is the 0% reduction in resazurin, 100 is the 100% reduction in resazurin, S is the sample value. Finally, the inhibitory concentrations for 50% inhibition of fungal growth (IC_50_) were calculated. The minimum inhibitory concentration (MIC) was calculated using Gompertz model and method for determining the MIC with nonlinear regression as described by Lambert and Pearson [61]. All experiments were performed in triplicate.

### 3.5. Anti-Candida Activity Assay

The panel of reference yeast strains: *Candida albicans* ATCC 102231, *Candida parapsilosis* ATCC 22019, *Candida glabrata* ATCC 90030 [62], *Candida auris* CDC B11903 and *Candida krusei* ATCC 14243 (ATCC-LGC Standards, Teddington, UK) were tested in this study. The series of two-fold dilutions of tested compounds was carried out in the sterile 96-well polystyrene microtitrate plates (Nunc, Roskilde, Denmark) obtaining concentrations from 1000 to 7.8 μg/mL in the medium. Simultaneously, the inocula of 24 h-cultures of yeasts in sterile physiological saline (0.5 McFarland standard density) were prepared and added to each well obtaining final density of 5 × 10^4^ CFU/mL. A positive (inoculum without tested compound) and negative (compound without inoculum) controls were added in each microplate. After incubation (35 °C, 24 h) the growth of yeasts was measured spectrophotometrically at 600 nm (BioTEK ELx808, Bio-Tek Instruments, Inc., Winooski, VT, USA). MICs were marked at the lowest concentration of the compound without the growth of yeasts. Then, 5 µL of the suspension from each well including controls was subcultured on the agar plates in order to determine the minimal fungicidal concentration (MFC). The plates were incubated at 35 °C for 24 h. The MFC were determined at the lowest concentration of compounds inhibiting the growth of microbes.

### 3.6. Docking Methodology

Docking was performed using the FlexX scoring function, as implemented in the LeadIT software package (LeadIT version 2.3.2; BioSolveIT GmbH, Sankt Augustin, Germany, 2017). The crystal structure of human O-GlcNAc transferase in complex with UDP-5S-GlcNAc and substrate peptide (keratin-7) (PDB id: 4XIF) and the crystal structure of yeast lanosterol 14-alpha demethylase complexed with voriconazole (PDB id: 5HS1) were downloaded from the Protein Data Bank (PDB) [63]. All steps of ligands and receptor preparation were carried out using default settings in BioSolveIT’s LeadIT software ((LeadIT version 2.3.2; BioSolveIT GmbH, Sankt Augustin, Germany, 2017). Chains A were selected, and the binding sites were defined to include residues within a 6.5 Å radius around the native ligand. Soft docking (allowing for a volume overlap of up to 100 Å^3^) was performed. The clash factor was set to 0.1. Other parameters were kept at default values. The conformation with the most favourable binding score was then selected for a detailed evaluation of binding site interactions. For 2D visualization, the PoseView dock widget, as implemented in LeadIT version 2.3.2 software, was used.

## 4. Conclusions

Developing novel lead structures for dermatophytosis treatment is currently an urgent need. We investigated the antidermatophytic activity of a series of imidazole-thiosemicarbazides against the three genera *Trichophyton*, *Microsporum*, and *Chrysosporium*. Our results show potent in vitro activity of the methyl (**1**–**3**) and the nitro (**5**, **6**) compounds against *T. tonsurans* and the *m*-bromine **7** and the *m*-iodine **8** analogues against *M. canis.* Furthermore, an in silico study elucidated their interactions in the active sites of both α-keratin and lanosterol-14-α demethylase; an enzyme which is the key action mechanism region of azole antifungal drugs, as well as luliconazole; and imidazole-based antifungal agent with a broad spectrum of activity against various species of fungi, majorly dermatophytes. A combined keratinophilic effect along with the interactions at the azole drug-target site were predicted, making these compounds suitable lead structures for further studies.

In our laboratory, in vitro studies using the zebrafish (Danio rerio) model are currently being conducted to comprehensively evaluate the safety, efficacy, and toxicity of IMI-TSCs. This model offers a valuable platform for high-throughput screening and real-time assessment of drug effects in a whole-organism context, enabling efficient identification of promising candidates for further development. If results are positive, specialized formulations—including lipid-based gels and film-forming gels, which provide convenient application and prolonged drug availability on the skin surface—are planned, along with topical formulations featuring advanced delivery systems such as liposomes, nanoemulsions, and microemulsions. These formulations enhance drug penetration, retention time, stability, and release profiles to improve effectiveness against dermatophytes.

## Figures and Tables

**Table 1:** In vitro antidermatophytic activity of the target compounds ^a^.

		MIC (μg/mL; [μM])							
	Compound	*T. ton.*	*T. rub.*	*T. men.*	*M. can.*	*A. ker.*	*Ch. que.*	*A. pan.*	*Ch. tro.*
**1**	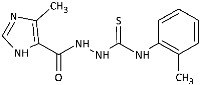	2.93 [10.14]	>289.36 [>1000]	>289.36 [>1000]	>289.36 [>1000]	>289.36 [>1000]	>289.36 [>1000]	>289.36 [>1000]	127.19 [>439.56]
**2**	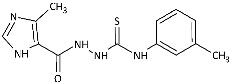	1.43 [4.94]	>289.36 [>1000]	>289.36 [>1000]	>289.36 [>1000]	>289.36 [>1000]	70.75 [244.51]	177.27 [612.65]	162.81 [562.67]
**3**	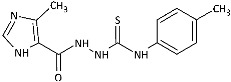	<0.29 [<1]	>289.36 [>1000]	>289.36 [>1000]	>289.36 [>1000]	>289.36 [>1000]	153.45 [530.32]	133.58 [461.65]	136.75 [472.61]
**4**	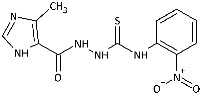	98.81 [308.46]	78.00 [243.50]	149.35 [466.23]	147.45 [460.30]	>320.33 [>1000]	42.28 [132.00]	41.64 [130.00]	146.28 [456.66]
**5**	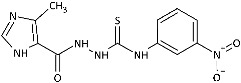	1.59 [4.96]	>320.33 [>1000]	>320.33 [>1000]	>320.33 [>1000]	>320.33 [>1000]	38.86 [121.31]	36.48 [113.87]	131.01 [409.00]
**6**	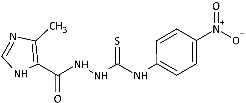	3.65 [11.40]	73.38 [229.07]	>320.33 [>1000]	>320.33 [>1000]	n.a.	39.41 [123.02]	49.82 [155.54]	52.69 [164.48]
**7**	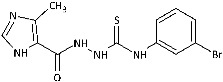	73.46 [207.39]	>354.23 [>1000]	271.58 [766.70]	7.38 [20.83]	>354.23 [>1000]	21.86 [61.71]	43.69 [123.34]	169.62 [478.86]
**8**	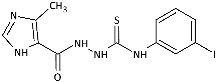	15.15 [37.77]	>401.23 [>1000]	165.51 [412.51]	3.87 [9.64]	>401.23 [>1000]	24.38 [60.76]	46.81 [116.67]	86.35 [215.21]
**9**	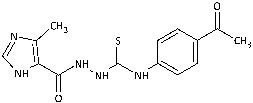	13.65 [43.02]	144.82 [456.31]	>317.37 [>1000]	136.05 [428.69]	n.a.	39.56 [124.64]	76.84 [242.11]	67.71 [213.35]
**10**	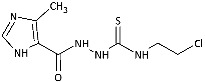	n.a.	n.a.	n.a.	n.a.	n.a.	n.a.	n.a.	n.a.
**11**	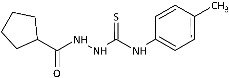	122.77 [442.61]	197.98 [713.75]	>277.39 [>1000]	152.26 [548.91]	141.44 [509.91]	143.72 [518.12]	170.93 [616.21]	3.68 [13.25]
**12**	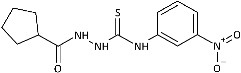	226.14 [733.38]	>308.36 [>1000]	>308.36 [>1000]	>308.36 [>1000]	>308.36 [>1000]	>308.36 [>1000]	>308.36 [>1000]	3.98 [12.91]
**13**	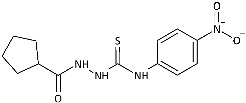	68.66 [222.66]	152.46 [494.44]	278.36 [902.71]	163.24 [529.40]	108.05 [350.41]	149.46 [484.71]	170.25 [552.11]	1.61 [5.23]
**14**	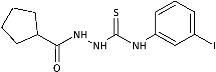	225.41 [579.09]	283.05 [727.15]	>389.26 [>1000]	>389.26 [>1000]	457.02 [1174.09]	355.12 [912.31]	>389.26 [>1000]	6.08 [15.61]
	**AMB**	4.00 [3.70]	4.00 [3.70]	4.00 [3.70]	8.00 [7.39]	4.00 [3.70]	nt	nt	nt
	**KCZ**	0.50 [0.27]	2.00 [1.06]	1.00 [0.53]	1.00 [0.53]	1.00 [0.53]	nt	nt	nt

^a^ Abbreviations: MIC, minimal inhibitory concentration; *T. ton.*, *Trichophyton tonsurans* CBS 118.65; *T. rub.*; *Trichophyton rubrum* CBS 120358; *T. men.*, *Trichophyton mentagrophytes* (*Trichophyton interdigitale*) CBS 124408; *M. can.*, *Microsporum canis* CBS 11348, *A. ker*., *Aphanoascus keratinophilus* (*Chrysosporium keratinophilum*) CBS 104, *Ch. que.*, *Chrysosporium queenslandicum* CBS 280.7762, *A. pan*., *Arthroderma pannicola* (*Chrysosporium pannicola*) CBS 116.63; *Ch. tro.*, *Chrysosporium tropicum* CBS 171.62; n.a.—no activity at highest tested concentration; nt—not tested; AMB—amphotericin B; KCZ—ketoconazole.

**Table 2:** The inhibitory concentration (IC_50_) and selectivity index (SI) of the target compounds against dermatophytes ^a^.

	IC_50_ [μM]; SI							
Compound	*T. ton.*	*T. rub.*	*T. men.*	*M. can.*	*A. ker.*	*Ch. que.*	*A. pan.*	*Ch. tro.*
**1**	[<1] SI > 996	[580.09] SI = 1.7	[55.14] SI = 18.1	[491.58] SI = 2.0	[>1000] SI < 1	[>1000] SI < 1	[460.47] SI = 2.2	[52.54] SI = 19.0
**2**	[10.14] SI = 17.1	[>1000] SI < 1	[>1000] SI < 1	[>1000] SI < 1	[>1000] SI < 1	[>1000] SI < 1	[>1000] SI < 1	[>439.56] SI < 1
**3**	[<1] SI > 361	[>1000] SI < 1	[32.07] SI = 11.3	[361.91] SI < 1	[>1000] SI < 1	[224.91] SI = 1.6	[305.70] SI = 1.2	[44.77] SI = 8.1
**4**	[2.05] SI = 276.5	[185.69] SI = 3.1	[415.33] SI = 1.4	[339.15] SI = 1.7	[>1000] SI < 1	[90.55] SI = 6.3	[92.77] SI = 6.1	[39.72] SI = 14.3
**5**	[<1] SI > 440	[>1000] SI < 1	[344.69] SI = 1.3	[269.64] SI = 1.6	[>1000] SI < 1	[78.52] SI = 5.6	[74.23] SI = 5.9	[76.05] SI = 5.8
**6**	[<1] SI > 682	[149.69] SI = 4.6	[>1000] SI < 1	[625.89] SI = 1.1	n.a.	[83.06] SI = 8.2	[100.39] SI = 6.8	[44.77] SI = 15.3
**7**	[<1] SI > 846	[>1000] SI < 1	[127.41] SI = 6.6	[11.06] SI = 76.6	[>1000] SI < 1	[38.17] SI = 22.2	[68.34] SI = 12.4	[111.99] SI = 7.6
**8**	[<1] SI > 213	[489.32] SI < 1	[66.49] SI = 3.2	[7.21] SI = 29.6	[619.86] SI < 1	[35.39] SI = 6.0	[46.24] SI = 4.6	[82.51] SI = 2.6
**9**	[<1] SI > 650	[419.46] SI = 1.6	[>1000] SI < 1	[404.29] SI = 1.6	n.a.	[84.81] SI = 7.7	[150.86] SI = 4.3	[42.07] SI = 15.5
**11**	[65.87] SI = 7.0	[510.74] SI < 1	[225.16] SI = 2.1	[390.66] SI = 1.2	[208.88] SI = 2.2	[380.97] SI = 1.2	[393.09] SI = 1.2	[<1] SI > 463
**12**	[216.97] SI = 2.8	[>1000] SI < 1	[563.64] SI = 1.1	[>1000] SI < 1	[398.01] SI = 1.5	[>1000] SI < 1	[>1000] SI < 1	[2.51] SI = 245.6
**13**	[36.58] SI = 10.3	[447.71] SI < 1	[66.50] SI = 5.7	[280.86] SI = 1.3	[120.08] SI = 3.2	[329.15] SI = 1.1	[283.14] SI = 1.1	[<1] SI > 378
**14**	[206.11] SI < 1	[538.26] SI < 1	[86.77] SI = 1.4	[>1000] SI < 1	[420.92] SI < 1	[306.34] SI < 1	[>1000] SI < 1	[1.16] SI = 101.4

^a^ Abbreviations: IC_50_—the concentration required for 50% inhibition of fungal growth; SI—selectivity index calculated by CC_30_/IC_50_. CC_30_ values were taken from refs. [28,42]; *T. ton.*, *Trichophyton tonsurans* CBS 118.65; *T. rub.*; *Trichophyton rubrum* CBS 120358; *T. men*., *Trichophyton mentagrophytes* (*Trichophyton interdigitale*) CBS 124408; *M. can*., *Microsporum canis* CBS 11348, *A. ker*., *Aphanoascus keratinophilus* (*Chrysosporium keratinophilum*) CBS 104, *Ch. que*., *Chrysosporium queenslandicum* CBS 280.7762, *A. pan*., *Arthroderma pannicola* (*Chrysosporium pannicola*) CBS 116.63; *Ch. tro*., *Chrysosporium tropicum* CBS 171.62.

**Table 3:** In vitro antifungal activity [μg/mL] of the target compounds against *Candida* spp. ^a^.

	*C. albicans*		*C. glabrata*		*C. auris*		*C. krusei*		*C. parapsilosis*	
Compound	MIC	MBC	MIC	MBC	MIC	MBC	MIC	MBC	MIC	MBC
**1**	>1000	>1000	>1000	>1000	500	1000	>1000	>1000	500	>1000
**2**	1000	1000	1000	>1000	250	500	1000	1000	500	1000
**3**	1000	1000	1000	>1000	250	500	1000	>1000	250	1000
**4**	>1000	>1000	>1000	>1000	250	250	1000	>1000	250	1000
**5**	500	>1000	1000	1000	250	500	1000	1000	250	1000
**6**	500	500	1000	>1000	62.5	125	500	500	250	500
**7**	1000	>1000	1000	>1000	500	1000	1000	>1000	500	1000
**8**	500	>1000	>1000	>1000	500	>1000	>1000	>1000	>1000	>1000
**9**	1000	1000	1000	>1000	250	250	1000	1000	500	1000
**Nystatin**	0.24	0.48	0.48	0.48	0.48	0.48	0.24	0.24	0.24	0.48

^a^ Abbreviations: MIC, minimal inhibitory concentration; MBC, minimal bactericidal concentration; *Candida albicans* ATCC 102231 [48]; *Candida glabrata* ATCC 66032 [49]; *Candida auris* CDC B11903 [50]; *Candida krusei* ATCC 14243 [51]; *Candida parapsilosis* ATCC 22019 [52].

**Table 4:** Scores of top poses of IMI-TSCs **1**–**9** docked to α-keratin (PDB ID 4XIF) and lanosterol-14-α demethylase (PDB ID 5HS1).

	1	2	3	4	5	6	7	8	9	NI *
α-keratin	−36.4	−39.2	−37.8	−43.3	−49.7	−38.7	−39.2	−38.5	−38.7	−58.9
lanosterol−14-α demethylase	−32.1	−33.5	−28.7	−33.8	−33.8	−38.2	−30.2	−29.0	−33.0	−27.5

* NI—native inhibitors.

## Data Availability

Dataset available on request from the authors.
